# Whole-genome association study of antibody response to Epstein-Barr virus in an African population: a pilot

**DOI:** 10.1017/gheg.2017.16

**Published:** 2017-11-27

**Authors:** N. Sallah, T. Carstensen, K. Wakeham, R. Bagni, N. Labo, M. O. Pollard, D. Gurdasani, K. Ekoru, C. Pomilla, E. H. Young, S. Fatumo, G. Asiki, A. Kamali, M. Sandhu, P. Kellam, D. Whitby, I. Barroso, R. Newton

**Affiliations:** 1 Department of Human Genetics, Wellcome Trust Sanger Institute, Hinxton, UK; 2 Department of Virus Genomics, Wellcome Trust Sanger Institute, Hinxton, UK; 3 Department of Medicine, University of Cambridge, Cambridge, UK; 4 MRC/Uganda Virus Research Institute, Uganda Research Unit on AIDS, Entebbe, Uganda; 5 Institute of Cancer Sciences, University of Glasgow, Glasgow, UK; 6 Protein Expression Lab, Cancer Research Technology Program, Leidos Biomedical Research, Frederick National Laboratory for Cancer Research, Frederick, MD, USA; 7 Viral Oncology Section, Aids and Cancer Program, Leidos Biomedical Research, Frederick National Laboratory for Cancer Research, Frederick, MD, USA; 8 H3Africa Bioinformatics Network (H3ABioNet) Node, National Biotechnology Development Agency (NABDA), Federal Ministry of Science and Technology (FMST), Abuja, Nigeria

**Keywords:** Africa, Epstein-Barr virus, genomics, immunity, infectious disease

## Abstract

Epstein Barr virus (EBV) infects 95% of the global population and is associated with up to 2% of cancers globally. Immunoglobulin G (IgG) antibody levels to EBV have been shown to be heritable and associated with developing malignancies. We, therefore, performed a pilot genome-wide association analysis of anti-EBV IgG traits in an African population, using a combined approach including array genotyping, whole-genome sequencing and imputation to a panel with African sequence data. In 1562 Ugandans, we identify a variant in *human leukocyte antigen* (*HLA*)-*DQA1*, rs9272371 (*p* = 2.6 × 10^−17^) associated with anti-EBV nuclear antigen-1 responses. Trans-ancestry meta-analysis and fine-mapping with European-ancestry individuals suggest the presence of distinct *HLA* class II variants driving associations in Uganda. In addition, we identify four putative, novel, very rare African-specific loci with preliminary evidence for association with anti-viral capsid antigen IgG responses which will require replication for validation. These findings reinforce the need for the expansion of such studies in African populations with relevant datasets to capture genetic diversity.

## Introduction

Epstein Barr virus (EBV) is a common human herpesvirus infecting 95% of the global adult population. Following primary infection, often in childhood, EBV establishes a latent infection in B cells, allowing virus persistence in the face of an active immune system. EBV can reactivate from latency and enter the lytic cycle allowing viral replication and transmission. The majority of people live with EBV infection with the absence of clinical symptoms. However, EBV causes the self-limiting condition, Infectious Mononucleosis and up to 2% of cancers, including Burkitt's lymphoma, Hodgkin's lymphoma, nasopharyngeal carcinoma and some gastric cancers [1, 2]. It is also thought to be a risk factor for the development of autoimmune diseases such as systemic lupus erythematosus, rheumatoid arthritis and multiple sclerosis [3, 4].

EBV infection induces a strong cell-mediated and humoral immune response which actively contains EBV replication in healthy individuals. Antibodies against EBV nuclear antigen-1 (EBNA-1) reflect infection history, whilst those against viral capsid antigen (VCA) reflect viral reactivation and together are widely used as markers to study the latent and lytic stages of infection, respectively. Early life infection and high antibody titres have been strongly linked to the development of certain cancers [2, 5–7]. In a single individual, antibody titres have been found to remain fairly constant throughout life in the absence of immunosuppression or intense stress [6]. In addition, inter-individual variability in IgG responses to EBNA-1 and VCA has been found to be a 32–48% heritable trait [8–10] and thus is suggestive of host genetic influence.

While EBV has been extensively studied, the host genetics underpinning potential disease outcome are still unclear [11], particularly in Africa. Recent genetic association studies in Mexican American, and European ancestry population cohorts have reported variants in the human leucocyte antigen (*HLA*) class II region of the major histocompatibility complex on chromosome 6p21.3, contributing to variability in responses to EBNA-1 [9, 12]. No genome-wide association studies (GWASs) have been done for anti-VCA IgG responses. With less than 5% of GWASs conducted in African populations [13, 14], the contribution of human genetic variation to disease traits in such diverse populations remains largely uncharacterized. We aim to bridge the gap in understanding host genetic factors that contribute to EBV immune response serological traits in an African population cohort.

Here, we present a pilot study describing the first genome-wide association analysis performed for anti-EBV IgG traits in an African population. We highlight the combination of whole-genome sequencing and imputing genotypes to a panel with additional African sequence data to aid discovery of low-frequency and population-specific variants. We replicate variants in the *HLA* class II region contributing to anti-EBNA IgG response levels and also perform trans-ethnic meta-analysis and fine-mapping of EBNA-1 IgG traits with an additional cohort of European ancestry, revealing distinct variants driving associations in the two populations. Finally, we identify four potentially novel, rare loci that are African-specific with preliminary evidence of association with anti-VCA IgG serostatus, warranting replication in larger sample sizes.

## Methods

### Samples and ethics

The general population cohort (GPC) is a community-based open cohort study originally established in 1989, by the UK Medical Research Council and the Uganda Virus Research Institute, in the Kalungu District, south-western Uganda, to examine prevalence, incidence, risk factors and trends of infection with the human immunodeficiency virus (HIV) in a rural African population [15]. Data are collected through an annual census, health questionnaire and include blood specimens for the serological survey, details of sexual behaviour, medical, socio-demographic and geographic factors. As part of a larger investigation of oncogenic infections in the GPC, we measured antibodies against EBV from a cross-sectional sample of people at three-time points between 1990 and 2008. The sample was age and sex-stratified to provide a 1:1 sex ratio and to increase the proportion of participants >15 years old. Of the original ~9000 people tested, we were able to link EBV phenotype results from 1570 people (Mean age ± s.d. = 34 ± 19.6 years, 54% female) to the genetic data generated from samples collected from the GPC in 2011. Informed consent was obtained for genetic testing from participants either with a signature or a thumbprint if the individual was unable to write. The study was approved by the Uganda Virus Research Institutes, Research Ethics committee (UVRI-REC) (Ref. GC/127/10/10/25), the Uganda National Council for Science and Technology (UNCST), and the UK National Research Ethics Service, Research Ethics Committee (UK NRES REC) (Ref. 11/H0305/5).

### Serology

We quantified mean fluorescence Intensities (MFI) of IgG antibodies to EBNA-1 and VCA using multiplex serology on the Luminex platform based on glutathione-S-transferase (GST) fusion capture immunosorbent assays combined with fluorescent bead technology [16]. In this study, 94% of individuals were categorized as seropositive based on detectable IgG MFI >519 and/or >165 cutoffs for EBNA-1 and VCA, respectively.

### Genotyping, imputation and whole genome sequencing

5000 GPC samples were densely genotyped on the Illumina HumanOmni 2.5 M BeadChip array and we then imputed additional variants into the genotype chip dataset using a merged 1000 Genomes phase 3 [17], African genome variation project [18] and UG2G (Uganda 2000 Genomes) (Gurdasani *et al.* in submission) reference panel in IMPUTE2 [19]. Whole genome sequencing was performed on 2000 samples with 100 base paired-end sequencing at 4× coverage on the Illumina HiSeq 2000 platform following the manufacturer's protocol (Gurdasani *et al.* in submission).

### Quality control

Stringent variant and sample quality control (QC) filtering were performed. Low-quality variants that mapped to multiple regions within the human genome or did not map to any region, and duplicate variants genotyped on the chip were removed. We excluded samples with a call rate <97% and heterozygosity >3 s.d. from the mean, discordant genetic sex and reported sex, and sites deviating from Hardy Weinberg equilibrium (*p* < 10^−8^). Following imputation, we only included high-quality sites (info score >0.3 and *r*^2^ > 0.6) with minor allele frequency (MAF) ⩾0.5%. We also removed samples without matching phenotype and genotype or sequence data. Of the merged datasets, 343 samples had overlapping genotype and sequence variant calls for which a final concordance of 93.1% was achieved for all SNPs. The merged datasets post QC filtering resulted in 1562 samples with EBV phenotypes and ~17 M SNPs across the autosomes and X-chromosome for analyses.

### Principal components analysis (PCA)

PCA was performed using SMARTPCA in Eigensoft v4.2 with 1000 Genomes Project phase 3 and African Genome Variation Project populations as a reference panel. PCA was done including markers with MAF ⩾0.05 after linkage disequilibrium (LD) pruning (*r*^2^ = 0.5) using a sliding window approach with a window size of 200 Kb, sliding 5 SNPs sequentially.

### Heritability analyses

Narrow-sense heritability (*h*^2^) for anti-EBNA-1 IgG and anti-VCA IgG traits were estimated using a linear mixed model (LMM) in FaST-LMM with two random effects, one based on genetic effects and the other on environmental effects using spatial location [20] recorded as global position system (GPS) coordinates as a proxy for environmental effects.

### Association analyses

We conducted analyses for both quantitative antibody traits and discrete serostatus (i.e. presence/absence of antibody response) based on MFI cutoffs applying a linear or logistic regression model, respectively, in R. For anti-EBNA-1 IgG analysis age, sampling round, Hepatitis B virus and Hepatitis C virus status were adjusted for as significant covariates (online Supplementary Table S1). For anti-VCA IgG analysis Kaposi's sarcoma-associated virus and HIV statuses were also adjusted for in addition as significant covariates (online Supplementary Table S1). Residuals of MFI values used for analyses were transformed using inverse, rank-based normalization in R to ensure a standard normal distribution for the phenotypes and ascertained by visualization and Shapiro–Wilk test in R (online Supplementary Fig. S1). To control for cryptic relatedness and population structure within the GPC, the GWAS was performed using the standard mixed model approach in genome-wide efficient mixed-model association (GEMMA) [21]. A kinship matrix to define pairwise genetic relatedness among individuals was generated using pooled imputed genotypes and sequence data for all autosomes and X-chromosome using the *k* = 1 option in GEMMA. The data were LD pruned (*r*^2^ = 0.2) using dosages and a MAF threshold of 1% was applied. Genotyping or sequencing method was also adjusted for as additional covariates during analysis in GEMMA. To identify distinct SNPs, conditional analysis was performed in GEMMA. Each SNP within 1 MB of the lead association SNP was conditioned. If any SNP was statistically significant it was added stepwise onto the mixed model and analysed jointly, this was done until no SNPs with *p* < 5 × 10^−9^ remained. All SNPs remaining statistically significant were considered distinct association signals. For conditional analysis where genotype data was unavailable, association summary statistics were obtained and conditional analysis as described above was performed using genome-wide complex trait analysis (GCTA).

### Functional annotation of candidate variants

To functionally annotate our most significant associations we used the Ensembl Variant Effect Predictor (VEP) and the gene/tissue expression database (GTEx) to access data on expression quantitative trait loci (eQTLs) from tissues.

### Trans-ethnic meta-analysis and fine mapping

MANTRA was used to perform a genome-wide trans-ethnic meta-analysis for anti-EBNA IgG responses with association summary statistics of 1473 EBV seropositive individuals from our Ugandan GWAS combined with publically available data of 1000 Genomes imputed European ancestry GWAS from 2162 seropositive individuals, giving a total of 3635 individuals with ~4.6 million shared SNPs for analysis. The MANTRA approach leverages differences in LD structures across populations to account for differences in genetic architecture and accommodates heterogeneity of allelic effects between distantly related populations within a Bayesian partition framework [22]. To determine statistical significance, we used a threshold of log_10_ Bayes Factor (BF) >6, which is comparable with a *p* < 5 × 10^−8^, previously determined by Wang *et al.* [23]. Heterogeneity of allelic effect sizes was calculated using Cochran's *Q*-test for heterogeneity in METAL [24]. Using MANTRA results we generated 99% credible sets most likely to drive association signals and contain causal variants (or tagging unobserved causal variants) and compared fine-mapping intervals for each associated lead SNP by analysing the variants 500 kb up and downstream of the lead SNP in the Ugandan and combined Ugandan + European datasets. For this, posterior probabilities were calculated for SNPs and then ranked in decreasing order according to BF, proceeding down the rank until the cumulative posterior probability exceeded 99% as described previously [25, 26]. All SNPs ⩾0.99 were included in the credible sets.

## Results

### Assessing the genetic contribution to anti-EBV IgG response traits in a rural African cohort

To assess the contribution of human genetic variation on antibody responses to EBV we investigated 1570 individuals from a rural GPC [15] in south-western Uganda, and performed a GWAS combining whole-genome sequencing and dense genotyping data with imputation to a merged 1000 Genomes phase 3, African genome variation project (AGVP) [18] and UG2 G (Uganda 2000 Genomes) (Gurdasani *et al.* in submission) reference panel. In this cohort, 94% (1473/1570) of individuals were categorized as EBV seropositive (i.e. presence of detectable IgG response to EBNA-1 or VCA) based on mean fluorescence intensity (MFI) cutoffs [16]. Stringent sample and variant QC left 1562 individuals with EBV anti-EBNA-1 and anti-VCA IgG phenotypes and ~17 M SNPs across autosomal markers and X-Chromosome for analyses as detailed in the methods section. Homogeneity in the study population was ascertained by PCA using Eigensoft v4.2 [27] with AGVP populations as a reference panel (online Supplementary Fig. S2).

As previous studies have reported heritability of IgG responses to EBNA-1 at 37–43% and to VCA at 32–48% [8–10], we also explored the heritability of anti-EBV IgG serological traits in the GPC. In this study, our estimates of heritability for anti-EBNA-1 and anti-VCA IgG responses were 21.5% and 9.8%, respectively, suggesting a heritable component albeit lower. After correction for a shared environment, accounted for by GPS coordinates [20], our estimates were further reduced to 12% and 7.6%, respectively. To further investigate the genetic determinants of response to EBV infection in this population we conducted GWAS for each continuous antibody trait and discrete serostatus using a linear mixed model and kinship estimation in GEMMA [21]. This model accounted well for population structure and cryptic relatedness as shown by genomic inflation factor (*λ*) ~1.0 for all traits (online Supplementary Fig. S3). Correcting for multiple testing and accounting for the lower LD in African populations the genome-wide significance threshold was adjusted to *p* < 5 × 10^−9^ (Gurdasani *et al.* manuscript in submission).

### Genetic determinants of anti-EBNA-1 IgG response

**Fig. 1.**
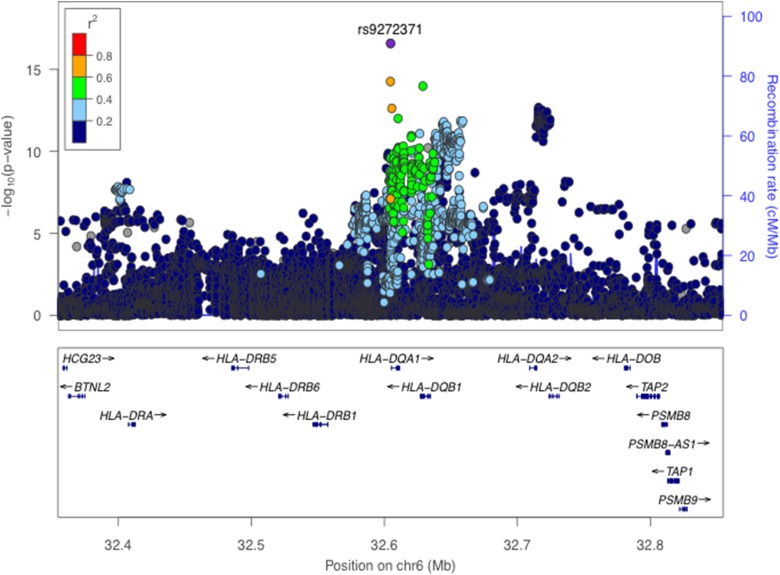
Regional association plot for anti-EBNA-1 IgG response levels in 1473 individuals. (Genome-Wide significance threshold = *p* < 5 × 10^−9^). The lead SNP rs9272371 (*p* = 2.6 × 10^−17^) located in an intron in *HLA-DRB1* on chromosome 6 is labelled and coloured in purple. LD (*r*^2^) was calculated based on the Ugandan SNP genotypes used in this study.

Consistent with previous findings, we identified significant associations for anti-EBNA-1 IgG antibody responses in the *HLA* class II region (Fig. 1 and online Supplementary Fig. S3). The C-allele at our lead SNP rs9272371 in *HLA-DQA1* (*p* = 2.6 × 10^−17^, *β* = −0.36) was associated with lower antibody response levels (Table 1), suggesting improved viral control and thus a protective effect on potential predisposition to disease. The same SNP in a European ancestry GWAS showed no evidence of significant association (*p* = 0.139) [12] (online Supplementary Table S2) and was absent in a Mexican American study [9]; as this may be owing to allelic heterogeneity or differences in LD structure in these populations, further investigation is needed to refine this signal (see below). The expression of 10 genes (*C4A, HLA-DQA1, HLA-DQB1-AS1, HLA-DQB1, HLA-DQB2, HLA-DRB1, HLA-DRB5, XXbac-BPG254F23.6, NOTCH4, HLA-DMA*) in 34 tissues were found to be affected by rs9272371 in the GTEx database. All of these genes are known to mediate immune function. rs9272371-C was significantly associated with a downregulation of expression of *HLA-DQA1* in all tissues including whole blood (eQTL *p* = 5.2 × 10^−36^, *β* = −0.75) and EBV transformed lymphocytes (eQTL *p* = 9 × 10^−12^, *β* = −0.94), which is consistent with the direction of our associations (Table 1).

**Table 1.** Summary of genome-wide significant association results in the GPC

Trait	Chr:Pos (b37)	SNP	Gene	Consequence	EA	EAF (%)	*p*, *β*_SNP_/OR (95% CI)
EBNA-1 QT	6:32604654	rs9272371	*HLA-DQA1*	Intronic	C	30.5	2.6 × 10^−17^, −0.36 (−0.26 to −0.42)
EBNA-1 Serostatus	6:32604654	rs9272371	*HLA-DQA1*	Intronic	C	30.5	3.5 × 10^−10^, 0.89 (0.86–0.93)
VCA Serostatus	2:43590060	rs183816209	*THADA*	Intronic	T	0.5	4.5 × 10^−9^, 0.59 (0.41–0.77)
VCA Serostatus	7:10280129	rs190139255	*–*	Intergenic	G	0.5	1.0 × 10^−9^, 0.57 (0.39–0.76)
VCA Serostatus	14:88403492	rs115256851	*GALC*	Intronic	C	1.1	6.9 × 10^−10^, 0.69 (0.57–0.81)
VCA Serostatus	17:64836303	rs114676416	*CACNG5*	Intronic	G	8.1	2.2 × 10^−9^, 0.86 (0.82–0.91)
EBV Multitrait	6:32604654	rs9272371	*HLA-DQA1*	Intronic	C	30.5	5.8 × 10^−21^, −0.36 (−0.27 to −0.44)

EA, effect allele; EAF, effect allele frequency; QT, quantitative trait.

### Trans-ethnic meta-analysis and fine mapping of anti-EBNA-1 IgG response

**Fig. 2.**
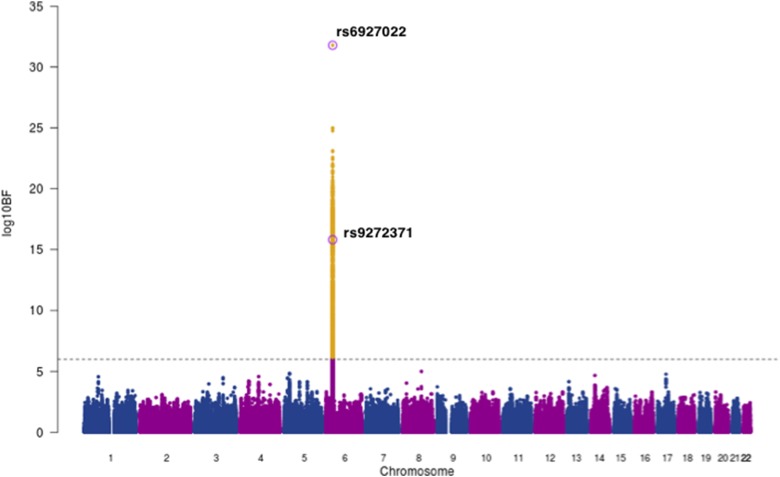
Trans-ethnic meta-analysis association plot for EBNA-1 IgG response levels in 3635 individuals of Ugandan and European Ancestry (EUR). Grey dashed line: threshold = log_10_ BF > 6. The lead SNPs for EUR (rs6927022) and Uganda (rs9272371) GWASs on chromosome 6 within the *HLA* region are labelled and circled in purple. Yellow: SNPs that meet the statistical significance threshold.

Next, we used MANTRA [22] to perform a genome-wide trans-ethnic meta-analysis for anti-EBNA IgG responses, with association summary statistics of 1473 EBV seropositive individuals from our Ugandan GWAS combined with 2162 seropositive individuals from the 1000 Genomes-imputed European ancestry GWAS [12], giving a total of 3635 individuals with ~4.9 million shared SNPs for analysis. We excluded genotype data from the Mexican American GWAS as the SNP density was not comparable. Using a threshold of log_10_ BF >6 [23] we found strong evidence of association in the *HLA* class II region with lead SNP rs6927022 (log_10_BF = 31.8) previously identified as the lead association SNP in the European ancestry study, whilst our Ugandan lead SNP rs9272371 (log_10_BF = 15.8) displayed heterogeneity in effect sizes in the two studies (*P*_*Q*_ = 3.56 × 10^−8^) (Fig. 2, Table 2). rs6927022 is similarly associated with the expression of nine out of the 10 genes affected by rs9272371. While rs6927022 was significant in our study (*p* = 2.01 × 10^−9^) and in moderate LD with our lead SNP rs9272371 (*r*^2^ = 0.32) (online Supplementary Fig S4), the association was markedly attenuated when conditioned on rs9272371 (*p*_cond_ = 0.0065) (online Supplementary Table S2). To further investigate whether the signals are distinct or partially tagging an un-typed functional variant contributing to both underlying association signals, we performed reciprocal conditional analysis of rs6927022 on our Uganda GWAS in GEMMA and also conditioned on our lead SNP rs9272371 in the European GWAS with association summary statistics using GCTA [28]. Both lead SNPs remained genome-wide significant in the respective cohorts after adjusting for the effect of the other (online Supplementary Table S2). Together, these findings suggest rs9272371 and rs6927022 are likely to be distinct variants in the *HLA* class II region, with a single signal in Europeans (rs6927022) and a signal mostly driven by rs9272371 in Uganda (online Supplementary Table S2). No other locus was found to be in association with anti-EBNA-1 IgG response.

**Table 2.** Loci with strong evidence of association with anti-EBNA-1 IgG levels after trans-ethnic meta-analysis of Ugandan and European ancestry GWASs

			Alleles	European ancestry (*N* = 2162)				Ugandan (*N* = 1473)				MANTRA EUR + UG (*N* = 3635)	
Lead SNP	Chr:Pos (b37)	Locus	Effect/Other	EAF	*β*	SE	*p*	EAF	*β*	s.e.	*p*	log_10_BF	*P*_Q_*
rs6927022^a^	6:32612397	HLA-DRB1	A/G	0.59	0.16	0.015	7.35 × 10^−26^	0.73	0.26	0.042	1.93 × 10^−09^	31.8	0.06
rs9272371^b^	6:32604654	HLA-DQA1	C/T	0.37	−0.02	0.015	0.14	0.30	−0.36	0.042	2.8 × 10^−17^	15.8	3.56 × 10^−8^

EAF, effect allele frequency; s.e., standard error.

a European (EUR) lead SNP.

b Ugandan (UG) lead SNP.

* *P*_*Q*_ – Cochran's *Q*-test for heterogeneity.

The availability of whole-genome sequence data and smaller LD blocks in African populations are advantageous for the refinement of genetic association signals. In line with this, using MANTRA results we generated 99% credible sets most likely to drive association signals and contain causal variants (or tagging unobserved causal variants) and compared fine mapping intervals for each associated lead SNP by analysing the variants 500 kb up and downstream of the lead SNP in the Ugandan and combined Ugandan + European datasets as described previously [25, 26]. This resulted in only one SNP in each credible set, rs6927022 for the Ugandan + European, and rs9272371 for the Ugandan GWAS respectively, further suggesting that rs6927022 does not fully drive associations in the Ugandan population.

### Genetic determinants of anti-VCA IgG serostatus

**Fig. 3.**
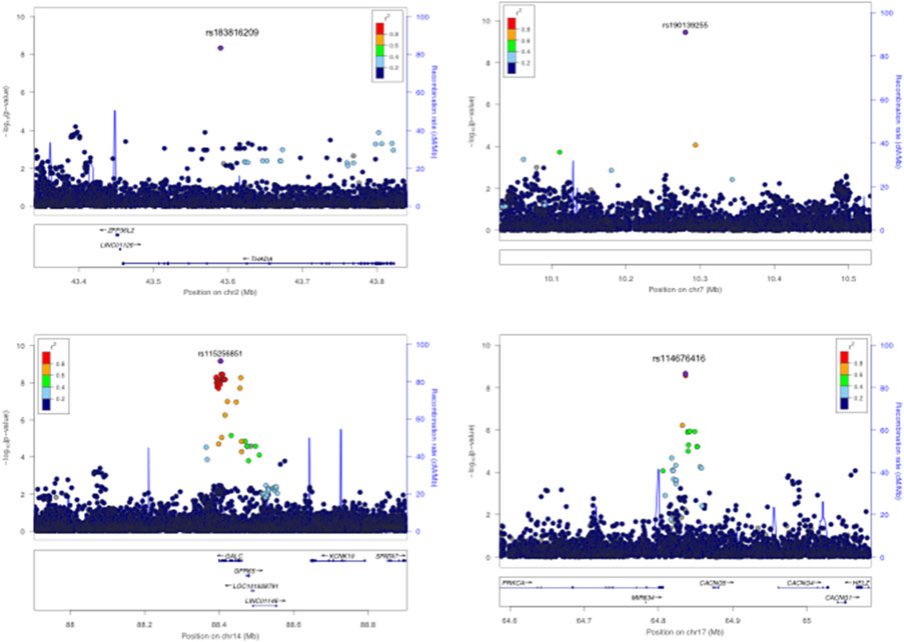
Regional association plots for VCA serostatus genome-wide significant associations, *N* = 1562, seropositive = 1344, seronegative = 217, threshold = *p* < 5 × 10^−9^. (*a*) Genome-wide significant association rs183816209 on Chromosome 2 in the *THADA* region (*p* = 4.5 × 10^−9^). (*b*) Genome-wide significant rs190139255 association on Chromosome 7 (4.0 × 10^−10^). (*c*) Genome-wide significant association rs115256851 on Chromosome 14 in the *GALC* region (6.8 × 10^−10^). (*d*) Genome-wide significant association rs114576416 on Chromosome 17 in the *CACNG5* region (2.2 × 10^−9^). The lead SNPs are labelled and coloured in purple. LD (*r*^2^) was calculated based on the Ugandan SNP genotypes used in this study.

For anti-VCA IgG response, 1344 individuals were categorized as seropositive and 218 individuals as seronegative based on VCA MFI cutoffs [16]. Using a case-control analysis for discrete serostatus (i.e. seropositive *v*. seronegative), we identified four potentially novel genome-wide significant associations (Fig. 3 and online Supplementary Fig. S5A). rs183816209-T (*p* = 4.5 × 10^−9^, OR = 0.59, MAF = 0.5%) an intronic variant in *THADA* on chromosome 2p21 (Fig. 3*a*), rs190139255-G (*p* = 4.0 × 10^−10^, OR = 0.57, MAF = 0.5%) an intergenic variant on chromosome 7p21.3 with the nearest gene a non-coding RNA *U3*, 17 kb upstream (Fig. 3*b*), rs115256851-C (*p* = 6.8 × 10^−10^, OR = 0.69, MAF = 1.1%) an intronic variant in *GALC* on chromosome 14q31.3 (Fig. 3*c*) and rs114576416-G (*p* = 2.2 × 10^−9^, OR = 0.86, MAF = 8.1%) an intronic variant in *CACGN5* on chromosome 17q24.2 (Fig. 3*d*). All lead SNPs passed variant filtering QC post imputation and non-reference alleles were concordant in individuals (*N* = 343) with overlapping genotype and sequence data, giving confidence in the accuracy of genotypes (online Supplementary Table S3). All SNPs were associated with seronegativity to VCA, potentially reflecting a lack of EBV lytic replication, and were low-frequency variants (Table 1). rs183816209 and rs115256851 were monomorphic in other 1000 Genomes phase 3 populations besides African ancestry, suggesting that they are African-specific. rs114676416 was also monomorphic in all populations except Africans and had MAF <1% in admixed Americans. rs190139255 had no allele frequency data reported in 1000 Genomes populations. No eQTL data were available for these SNPs in the gene/tissue expression database (GTEx) database. Quantitative analyses of anti-VCA IgG levels did not yield any genome-wide significant associations (online Supplementary Fig. S5B). A multivariate analysis combining anti-EBNA-1 and anti-VCA IgG phenotypes (*r*^2^ = 0.3) did not yield any additional genome-wide significant results (online Supplementary Fig. S6). No secondary associations were identified following conditional analyses on the lead SNPs for all traits.

## Discussion

In this study, we assessed the host genetic contribution on anti-EBV IgG responses in a rural African population cohort and highlight the utility of dense genotyping combined with whole-genome sequencing and imputation of genotypes to a combined reference panel with African sequence data to aid locus discovery and refinement of causal variants. As we are limited by sample size, particularly in African populations, to conduct well-powered GWASs for EBV-associated diseases such as Burkitt's Lymphoma, IgG response traits provide a good intermediate phenotype, indicating the strength of the humoral immune response and control of infection. EBV infection is nearly ubiquitous in Africa, with infection occurring early in childhood [7, 29, 30] and thus seronegativity based on cutoffs, which are arbitrarily determined most likely reflect a low-level immune response as opposed to lack of exposure to EBV. Previous studies have shown a correlation of IgG levels both with Burkitt's and Hodgkin's Lymphomas [5, 6, 31, 32], potentially predictive of disease risk.

It is interesting that despite the fact that both anti-EBV IgG traits display low heritability in this population after accounting for a shared environment, we still identify strong associations with SNPs contributing to variability in immune responses in African populations. This suggests that while genetic factors play a role in inter-individual variability in immune responses, environmental effects may have not been well accounted for by other studies, or differences in gene–environment interactions between populations result in different estimates. It is also a possibility that differences in assay/study design could contribute to differences in heritability estimates, however, in this setting, exposure to other pathogens are potentially strong cofactors influencing these traits and thus have been adjusted for in the study.

We successfully replicated association signals for anti-EBNA-1 IgG responses identified in individuals of Mexican American and European descent, and through trans-ethnic meta-analysis of European and African individuals in addition to fine-mapping identify distinct association signals in the *HLA* class II region. As a result of the complex LD structure in the HLA region, it is possible that both SNPs are tagging an underlying HLA allele, which we would have to explore further. Disentangling signals in the HLA region to pinpoint causal alleles is nontrivial owing to the poor representation of African ancestry data on HLA imputation reference panels that are heavily skewed towards European populations. In the European GWAS, Hammer and colleagues were able to achieve resolution of 4 digit classical *HLA* alleles and amino acids in *HLA-DRB1* through imputation using the Type 1 diabetes genetics consortium (T1DGC) Immunochip/HLA reference panel, which is predominantly European [12, 33]. *HLA* class II molecules present peptides to CD4+ T cells (T helper cells) eliciting both cell-mediated and antibody responses to control viral infection. EBV has also been found to use HLA class II molecules as a co-factor mediating entry into B cell lymphocytes [34, 35]. Given *HLA* haplotypes are highly polymorphic and display geographic variability, conducting host genetic studies in diverse populations will allow us to capture variation and understand its’ contribution to EBV immune control and disease.

Previously, no GWASs had been done for anti-VCA IgG responses and one linkage analysis had been performed without success in identifying statistically significant associations. For the first time, we have identified putative novel, African-specific genetic loci with evidence of association with anti-VCA IgG serostatus (Table 1). While two of the association signals, rs115256851 and rs114576416 appear more robust with MAFs >1% and multiple SNPs in the region highlighting evidence of association, the other two SNPs rs183816209 and rs190139255 show weaker evidence of association, with MAF ~0.5% and minimal or no SNPs in the region despite a high density of SNPs typed. Therefore, taking into account that the SNPs are rare, replication in larger sample sizes will be essential to validate these findings particularly as the majority (>90%) of individuals are infected with EBV (i.e. Cases) and thus the number of controls is relatively small.

In summary, the results of our pilot study substantiate the contribution of host genetic variation to EBV immune response and viral control. Our study reinforces the importance of studying diverse populations to uncover population-specific variants, differences in effect sizes and gene–environment interactions, which are known to vary significantly between European and non-European populations. A limitation at this stage is that with a small sample size we are underpowered to reliably identify rare genetic variants. Expanding these studies in African populations to include replication of the putative novel loci in larger sample sizes is key to validating our findings. In addition, the development of African resources such as an HLA imputation reference panel based on African genetic data and gene expression data will be crucial to be able to leverage approaches such as GWAS and refine our findings. While GWAS still remains a leading tool to identify variants, to follow up significant findings and gain biological insights, the development of pathway analysis tools with African populations also well represented would be necessary to reliably identify gene enrichments in pathways and protein interaction networks.

## Supplementary material

For supplementary material accompanying this paper visit http://dx.doi.org/10.1017/gheg.2017.16. click here to view supplementary material
