# Supplementary material for: Whole-genome association study of antibody response to Epstein-Barr virus in an African population: a pilot
Source: Glob Health Epidemiol Genom. 2017 Nov 27;2:e18. doi: 10.1017/gheg.2017.16 (PMC5870407; doi:10.1017/gheg.2017.16)
Supplement: Supplementary file 1 [file S2054420017000161sup001.pdf]

# Whole-Genome Association Study of Antibody Response to Epstein-Barr Virus in an African Population: A Pilot

## Supplementary Figures and Tables

**A**

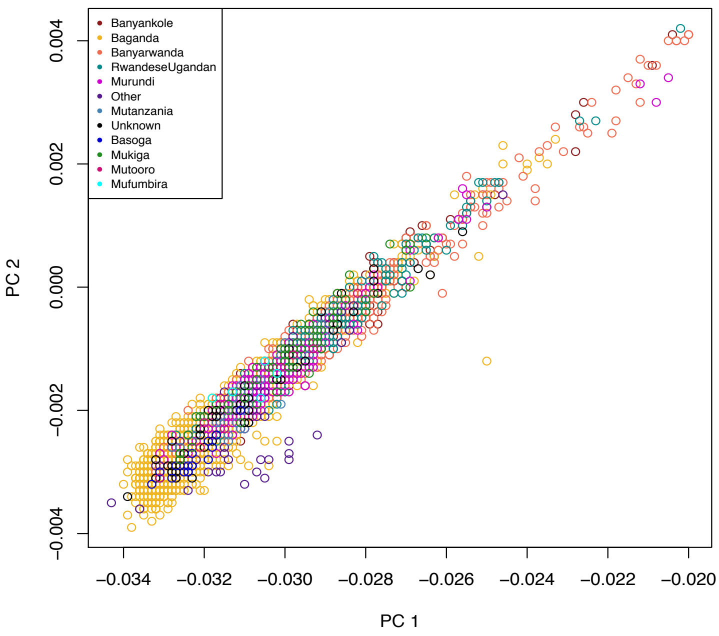


**B**

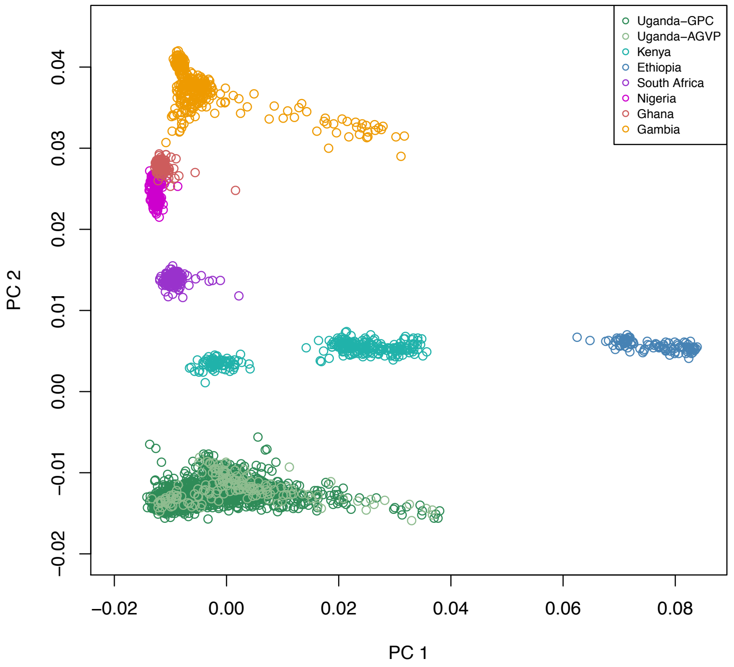


**Supplementary Fig. S1. PCA showing genetic population structure of A.** Individuals within the GPC ethnolinguistic groups in this study. No clear separation observed based on ethnolinguistic groups. **B**. Individuals in the GPC in the context of AGVP African populations. PC1 shows cline seen among East and West Africans. PC2 represents separation by

populations from different regions. Coloured circles represent respective ethnolinguistic groups or populations.

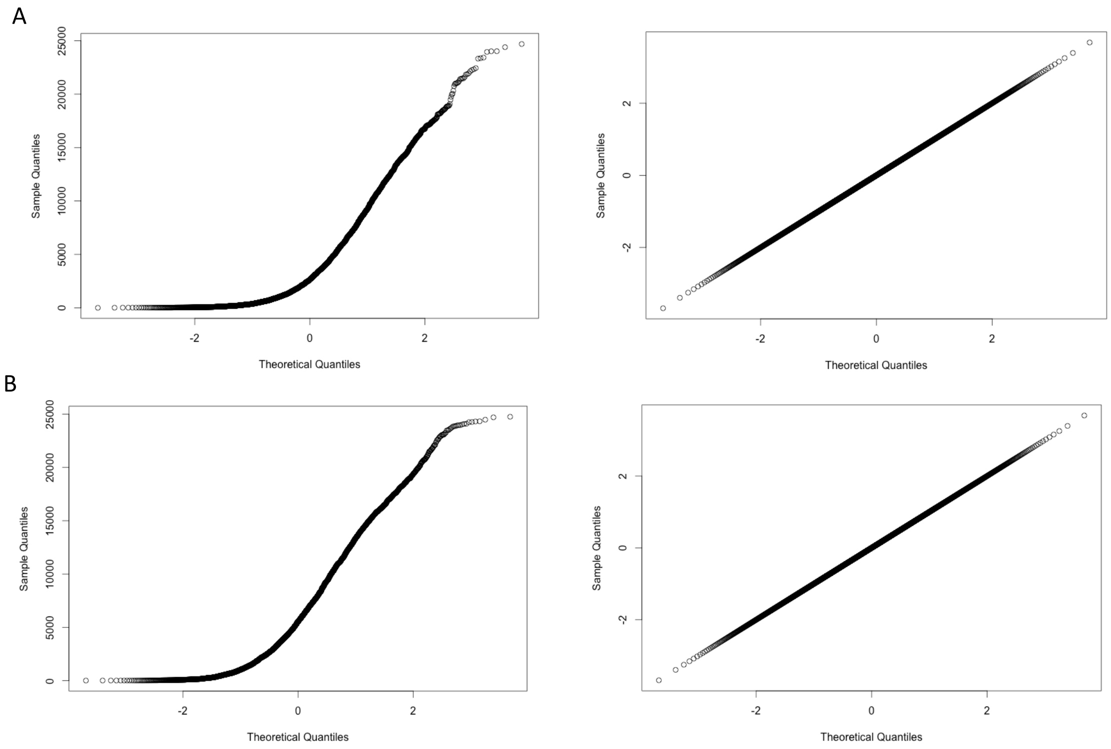


**Supplementary Fig S2. QQ-plots showing distribution of IgG antibody response MFI values.** Pre-transformation (left panel) and post-transformation (right panel), Shapiro-Wilk test used to assess normality. **A.** Distribution of Anti-EBNA-1 IgG $p_{untransformed}$= $2.2x10^{-16}$ and $p_{transformed}$=0.99. **B**. Distribution of Anti-VCA IgG $p_{untransformed}$=$2.2x10^{-16}$ and $p_{transformed}$= 0.99

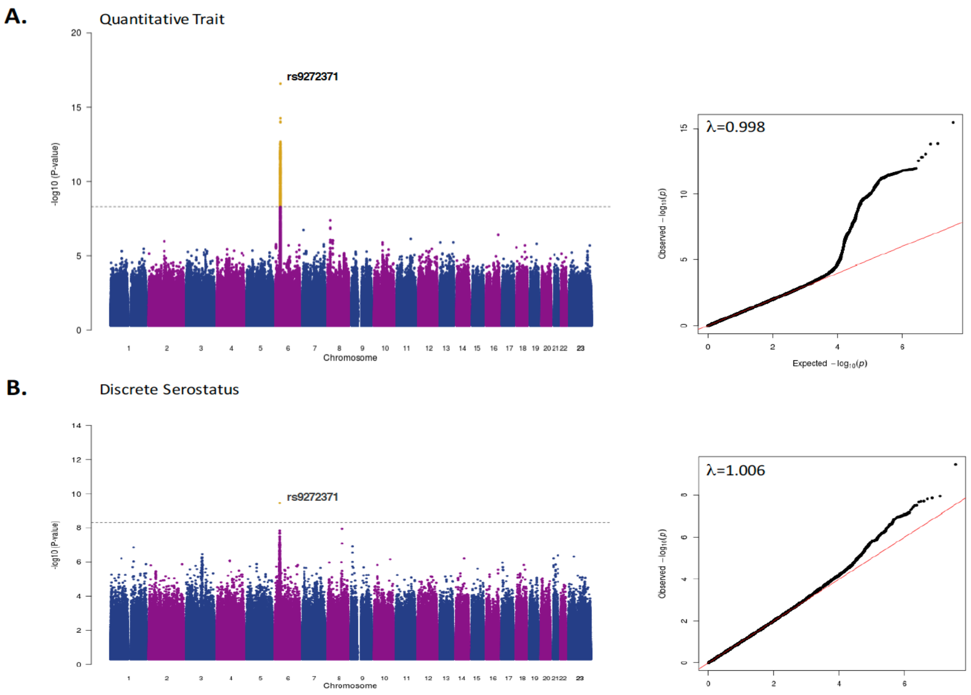


**Supplementary Figure S3. Genome Wide Association results of anti-EBNA-1 IgG response.** Manhattan Plots (Left Panel), grey dashed line: Genome wide significance threshold ($p<5x10^{-9}$) and QQ Plots (Right panel). **a.** Quantitative IgG response levels of EBV seropositive individuals (N=1473). **b.** Discrete Serostatus (Seropositive=1201, Seronegative=361). 23=X-Chromosome

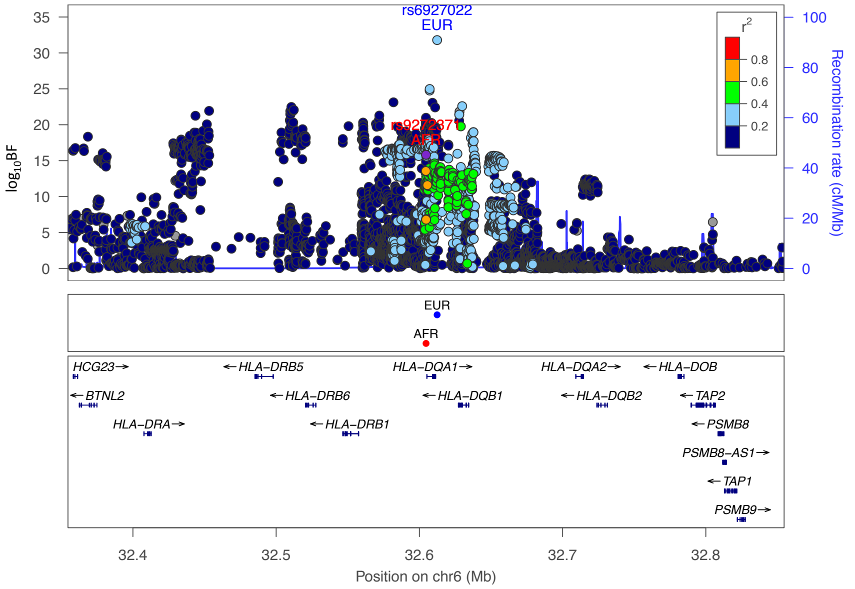


**Supplementary Figure S4. Trans ancestry fine mapping regional association plots for anti-EBNA-1 IgG levels in 3635 individuals (threshold= $log_{10}BF$ >6).** The Ugandan lead SNP (AFR) is labelled in red and coloured in purple. LD ($r^2$) was calculated based on SNP genotypes in the Ugandan dataset. The European (EUR) lead SNP is also labeled in blue.

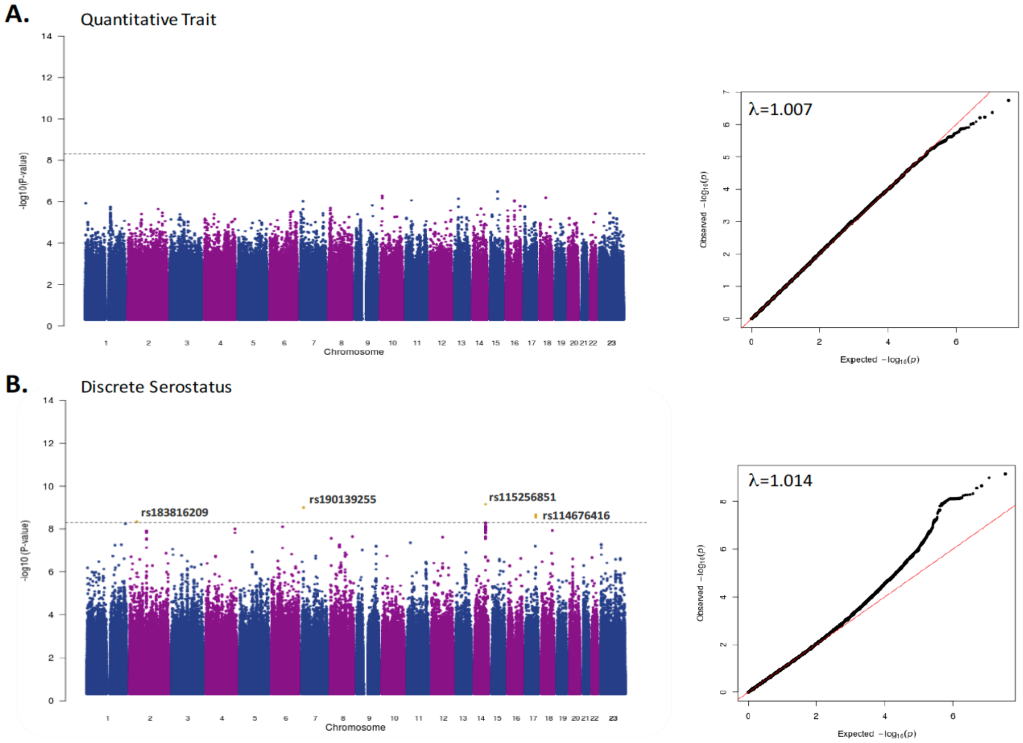


**Supplementary Figure S5. Genome Wide Association results of anti-VCA IgG response.** Manhattan Plots (Left Panel), Grey dashed line: Genome wide significance threshold ($p<5x10^{-9}$) and QQ Plots (Right panel). **a.** Quantitative IgG response levels of EBV seropositive individuals (N=1473). **b.** Discrete Serostatus (Seropositive=1344, Seronegative=218). 23=X-Chromosome

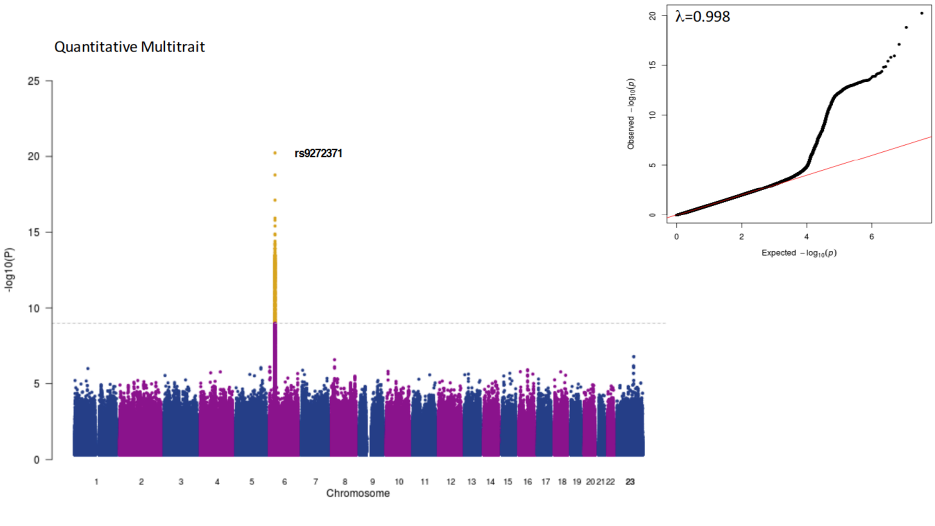


**Supplementary Figure S6. Multivariate Genome Wide Association results of anti-EBV IgG response levels**. Manhattan Plot (Left Panel) and QQ Plot (Right panel). N=1473. Grey dashed line: Genome wide significance threshold ($p<5x10^{-9}$). 23=X-Chromosome

**Supplementary Table S1. Covariates of IgG response levels for EBV infection**

| IgG | Age | Sex[a] | Sampling round[b] | KSHV Status[c] | HIV Status[c] | HBV Status[c] | HCV Status[c] |
|---|---|---|---|---|---|---|---|
| **EBNA-1** | **-0.009 ($4.4x10^{-6}$)** | -0.008 (0.91) | **0.020 ($2.0x10^{-2}$)** | 0.323 (0.02) | -0.036 (0.79) | **0.470 ($2.7 x10^{-4}$)** | **0.590 ($2.0x10^{-4}$)** |
| **VCA** | 0.002 (0.2) | 0.100 (0.1) | **0.020 ($2.7 x10^{-6}$)** | **0.479 ($1.6x10^{-4}$)** | **-0.680 ($3.5 x10^{-7}$)** | **0.580 ($1.2x10^{-6}$)** | **0.500 ($5.0x10^{-4}$)** |

All p-values in bold remain statistically significant after correcting for multiple testing using Bonferroni correction p<0.007.

[a] Positive regression coefficient relates to higher MFI/OD values in females than males.

[b] Positive regression coefficient relates to higher MFI/OD values in later sampling rounds.

[c] Positive regression coefficient relates to higher MFI/OD values in seropositive than seronegative individuals.

**Supplementary Table S2. Conditional analysis of lead Ugandan and European SNPs**

| | *p*(GWAS) | | Condition on rs9272371 *p*(cond) | | Condition on rs6927022 *p*(cond) | |
|---|---|---|---|---|---|---|
| **SNP** | **Uganda** | **EUR[11]** | **Uganda** | **EUR*** | **Uganda** | **EUR** |
| **rs9272371[a]** | $2.8x10^{-17}$ | 0.139 | - | - | $5.9x10^{-10}$ | 0.316 |
| **rs6927022[b]** | $1.93x10^{-09}$ | $7.35x10^{-26}$ | 0.0065 | $4.5x10^{-26}$ | - | - |

[a]Uganda Lead SNP

[b]EUR (European) Lead SNP

* Conditional analysis performed with association summary statistics in GCTA

**Supplementary Table S3. Non-reference allele concordance rate for SNPs associated with anti-VCA IgG serostatus**

| Chr:Pos (b37) | SNP | Allele | AF (%) | Matches | Mismatches | Concordance (%) |
|---|---|---|---|---|---|---|
| 2:43590060 | rs183816209 | T | 0.5 | 2 | 1 | 66.7 |
| 7:10280129 | rs190139255 | G | 0.5 | 3 | 0 | 100 |
| 14:88403492 | rs115256851 | C | 1.1 | 19 | 1 | 95.0 |
| 17:64836303 | rs114676416 | G | 8.1 | 53 | 4 | 91.4 |

AF=Allele Frequency
